# The commonness in immune infiltration of rheumatoid arthritis and atherosclerosis: Screening for central targets *via* microarray data analysis

**DOI:** 10.3389/fimmu.2022.1013531

**Published:** 2022-10-13

**Authors:** Zuoxiang Wang, Qingyue Xia, Wenxing Su, Mingyang Zhang, Yiyu Gu, Jialiang Xu, Weixiang Chen, Tingbo Jiang

**Affiliations:** ^1^ Department of Cardiology, The First Affiliated Hospital of Soochow University, Suzhou, China; ^2^ Department of Dermatology, The First Affiliated Hospital of Nanjing Medical University, Nanjing, China; ^3^ Department of Plastic and Burn Surgery, The Second Affiliated Hospital of Chengdu Medical College, China National Nuclear Corporation 416 Hospital, Chengdu, China

**Keywords:** atherosclerosis, rheumatoid arthritis, immune infiltration, hub genes, memory B cells, follicular helper T cells, γδT cells

## Abstract

**Background:**

Although increasing evidence has reported an increased risk of atherosclerosis (AS) in rheumatoid arthritis (RA), the communal molecular mechanism of this phenomenon is still far from being fully elucidated. Hence, this article aimed to explore the pathogenesis of RA complicated with AS.

**Methods:**

Based on the strict inclusion/exclusion criteria, four gene datasets were downloaded from the Gene Expression Omnibus (GEO) database. After identifying the communal differentially expressed genes (DEGs) and hub genes, comprehensive bioinformatics analysis, including functional annotation, co-expression analysis, expression validation, drug-gene prediction, and TF-mRNA-miRNA regulatory network construction, was conducted. Moreover, the immune infiltration of RA and AS was analyzed and compared based on the CIBERSORT algorithm, and the correlation between hub genes and infiltrating immune cells was evaluated in RA and AS respectively.

**Results:**

A total of 54 upregulated and 12 downregulated communal DEGs were screened between GSE100927 and GSE55457, and functional analysis of these genes indicated that the potential pathogenesis lies in immune terms. After the protein-protein interaction (PPI) network construction, a total of six hub genes (*CCR5, CCR7, IL7R, PTPRC, CD2*, and *CD3D*) were determined as hub genes, and the subsequent comprehensive bioinformatics analysis of the hub genes re-emphasized the importance of the immune system in RA and AS. Additionally, three overlapping infiltrating immune cells were found between RA and AS based on the CIBERSORT algorithm, including upregulated memory B cells, follicular helper T cells and γδT cells.

**Conclusions:**

Our study uncover the communal central genes and commonness in immune infiltration between RA and AS, and the analysis of six hub genes and three immune cells profile might provide new insights into potential pathogenesis therapeutic direction of RA complicated with AS.

## Introduction

Rheumatoid arthritis (RA) is a chronic systemic inflammatory disorder characterized by articular and extra-articular involvement, with a global prevalence of 0.24% (1, 2). RA patients were reported to have higher cardiovascular morbidity and mortality (3, 4). Atherosclerosis (AS) is defined as the formation of fibrofatty lesions in the artery wall, commonly found in the cardiovascular and cerebrovascular systems, that has been the major cause of disability and death worldwide (5, 6). Clinic evidence has shown that patients with RA had a 68% increased risk of myocardial infarction (MI), and the risk of developing silent MI was twofold higher in RA patients compared with normal (7, 8). Even in groups without a history of coronary heart disease, patients with RA had more coronary plaques and were more likely to form vulnerable plaques (7, 9, 10). The association between the two diseases has not been only reported from a clinical perspective. Nearly 10 years of research have also found that RA and AS have similar pathological processes and shared risk factors (11, 12), the most pivotal among which are chronic inflammation and immune activation (13, 14). Innate and adaptive immune system activation promotes higher cumulative inflammation that involves cytokines, immune cells, and non-immune cells, such as tumor necrosis factor-α (TNF-α) and interleukin-6 (IL-6) production, T and B cells activation, and the increase in epithelial cells, fibroblasts, and smooth muscle cell, etc. (11, 15, 16). In addition, chronic inflammation not only increases the traditional risk factors but also interacts with their mechanisms (11). The above-complicated biological process leads to endothelial dysfunction, arterial stiffness, and atherosclerotic plaque formation and progression, ultimately accelerating the atherogenic process in RA (12, 13, 17, 18). However, the exact biological pathway and molecular mechanism behind the above-mentioned biological process between RA and AS is still far from being elucidated. Meanwhile, some biological medications, such as anti-TNF medications and IL-1 inhibitors, have been reported to improve vascular function and reduce the risk of MI by easing the inflammatory burden to some extent (9, 19, 20). Hence, there is an urgent need to uncover more and exact biological targets in patients with RA to prevent AS occurrence and development.

Microarray technology is an effective analysis that is widely adopted to compare genes that are differentially expressed in biological models or patients under different diseases state (21, 22). Microarray technology is also used to better understanding gene association, mapping, expression, and linkage studies. At present, some articles have analyzed the RA or AS datasets from a single disease perspective, and dig out the potential targets and regulatory biological pathway of RA or AS respectively (21, 23). These studies provide new insights and research directions for single diseases of RA or AS, yet there is still a lack of studies to systematically explore the commonality of RA and AS and provide exact research directions. The common transcription signatures may indicate new insights into the common pathogenesis and immune mechanism of RA and AS. Hence, the purpose of this research was to uncover the biological mechanism and relevant immune pathway of RA complicated with AS, and more significantly, to explore the potential biomarkers and therapeutic directions of the two diseases. In this study, GSE55457 and GSE100927 were downloaded from the Gene Expression Omnibus (GEO) database to identify the communal differentially expressed genes (DEGs) between RA and AS. Subsequently, we construct the protein-protein interaction (PPI) network of communal DEGs to determine hub genes *via* STRING database and Cytoscape software. Comprehensive bioinformatics analysis, including functional annotation, co-expression analysis, expression validation, drug-gene prediction, and TF-mRNA-miRNA regulatory network construction, was performed to uncover the biological characteristic of hub genes. Additionally, we analyzed and compared the immune infiltration of RA and AS based on the CIBERSORT algorithm and evaluated the correlation between hub genes and infiltrating immune cells. Finally, a total of 66 communal DEGs, six hub genes, and three infiltrating immune cells that might indicate new insights into the relevant immune mechanisms and therapeutic directions of RA complicated with AS, were identified.

## Materials and methods

### Data source

GEO (http://www.ncbi.nlm.nih.gov/geo) is a microarray and high-throughput sequencing database created by NCBI (24). RA and AS were used as keywords to screen for qualified gene datasets based on strict inclusion/exclusion criteria. The inclusion criteria were as follows: (1) Sporadic RA or AS; (2) datasets containing patients and healthy controls and including the largest possible sample size; (3) the test specimens in datasets derived from human tissues. Exclusion criterium was: patients who participated in a clinical trial for drugs or other treatments. After filtering and comparing, four gene datasets were chosen from GEO based on strict inclusion/exclusion criteria. GSE55457 (13 RA patients and 10 controls) and GSE55235 (10 RA patients and 10 controls) were screened as RA datasets (25). Meanwhile, in the AS group, GSE100927 (69 AS human tissues and 35 controls) and GSE28829 (13 early atherosclerotic plaques and 16 advanced atherosclerotic plaques) were selected from GEO (26, 27). GSE55457 and GSE100927 were used to screen DEGs and perform Immune Infiltration analysis, while GSE55235 and GSE28829 were used for hub gene expression validation.

### Identification and enrichment analyses of overlapped DEGs

GEO2R (www.ncbi.nlm.nih.gov/geo/ge2r) is a network tool that works *via* the Limma package and GEOquery (28). DEGs of RA and AS were respectively identified using GEO2R with the condition of P-value < 0.05 and |logFC| > 1. After that, the overlap DEGs between RA and AS were detected and visualized by constructing VENN diagrams.

Based on the DAVID database (https://david.ncifcrf.gov/), Gene Ontology (GO) enrichment was conducted to analyze the biological characteristics of the overlap DEGs at the functional and molecular levels (29). The enrichment results are sorted separately by P-value and gene count in order to reveal the more meaningful biological processes. In addition, we performed pathway enrichment analysis of overlap DEGs from five pathway databases (KEGG PATHWAY, PID, BioCyc, Reactome and Panther) *via* network platform KOBAS 3.0 (30). P-value < 0.05 was considered statistically significant.

### PPI network construction, hub genes selection and analyses

A PPI network could reveal protein interactions and unearth the core protein genes. Upon the condition of interaction combined score > 0.4, the PPI network of overlap DEGs was constructed based on the STRING database (http://string-db.org) and visualized using Cytoscape software (31, 32).

CytoHubba is a Cytoscape plugin that could calculate the core protein genes of the PPI network (33). There are up to 12 algorithms in CytoHubba, all of which are already proven to be effective in screening hub genes. Five of the 12 algorithms were selected randomly in CytoHubba to calculate the top 10 core genes and determine the hub genes by taking the intersection of five algorithms running outcomes.

Metascape (https://metascape.org) platform was adopted here to perform functional annotation analysis of hub genes, and further pathway enrichment was performed by KOBAS 3.0 (34) from five above-mentioned pathway databases. Subsequently, we constructed and analyzed the co-expression network of hub genes *via* the GeneMANIA (http://www.genemania.org/) platform which is a reliable network tool for analyzing gene list function and exploring internal associations (35). Additionally, we predicted the drug-gene pairs of hub genes using DGIdb 3.0 (http://www.dgidb.org) database based on the condition that a predicted drug was FDA-approved (36). After the prediction, the network map of drug-gene pairs was visualized *via* Cytoscape software.

### Validation of hub gene expression

The expression of hub genes was verified in GSE55235 (10 RA patients and 10 controls) and GSE28829 (13 early atherosclerotic plaques and 16 advanced atherosclerotic plaques) respectively, and the comparison between the two sets of data was conducted with the T-test. A P value of < 0.05 was considered statistically significant.

### Construction of the TF-mRNA–miRNA regulatory network

Mirwalk is a credible database basically focused on miRNA-target interactions (37). In this research, we predicted the miRNA of hub genes *via* the Mirwalk database under the strict condition that the predicted interactions could be verified by experiments or other databases. Transcriptional Regulatory Relationships Unraveled by Sentence-based Text mining (TRRUST) database consisting of the target genes corresponding to TFs and the regulatory relationships between TFs, was adopted to predict the TFs that regulate the hub genes (38). After the prediction, the regulatory network of TF-mRNA-miRNA was constructed and visualized using Cytoscape software.

### Evaluation of immune cell infiltration and correlation analysis

On the ground of the CIBERSORT algorithm, we analyzed the relative percentage of 22 immune cell subpopulations in AS and control samples from the GSE100927. Besides, violin diagrams were generated to compare and visualize the difference in the immune cell between AS and control *via* the ggplot2 package. Consistent with the GSE100927, the percentage of immune cells and the difference between RA and control from GSE55457 were analyzed and visualized in the same way.

Person correlation analysis was carried out on hub genes and infiltrating immune cells by the ggstatsplot package, and the results were visualized by ggplot2 package.

## Result

### Identification and enrichment analyses of overlapped DEGs

**Figure 1:**
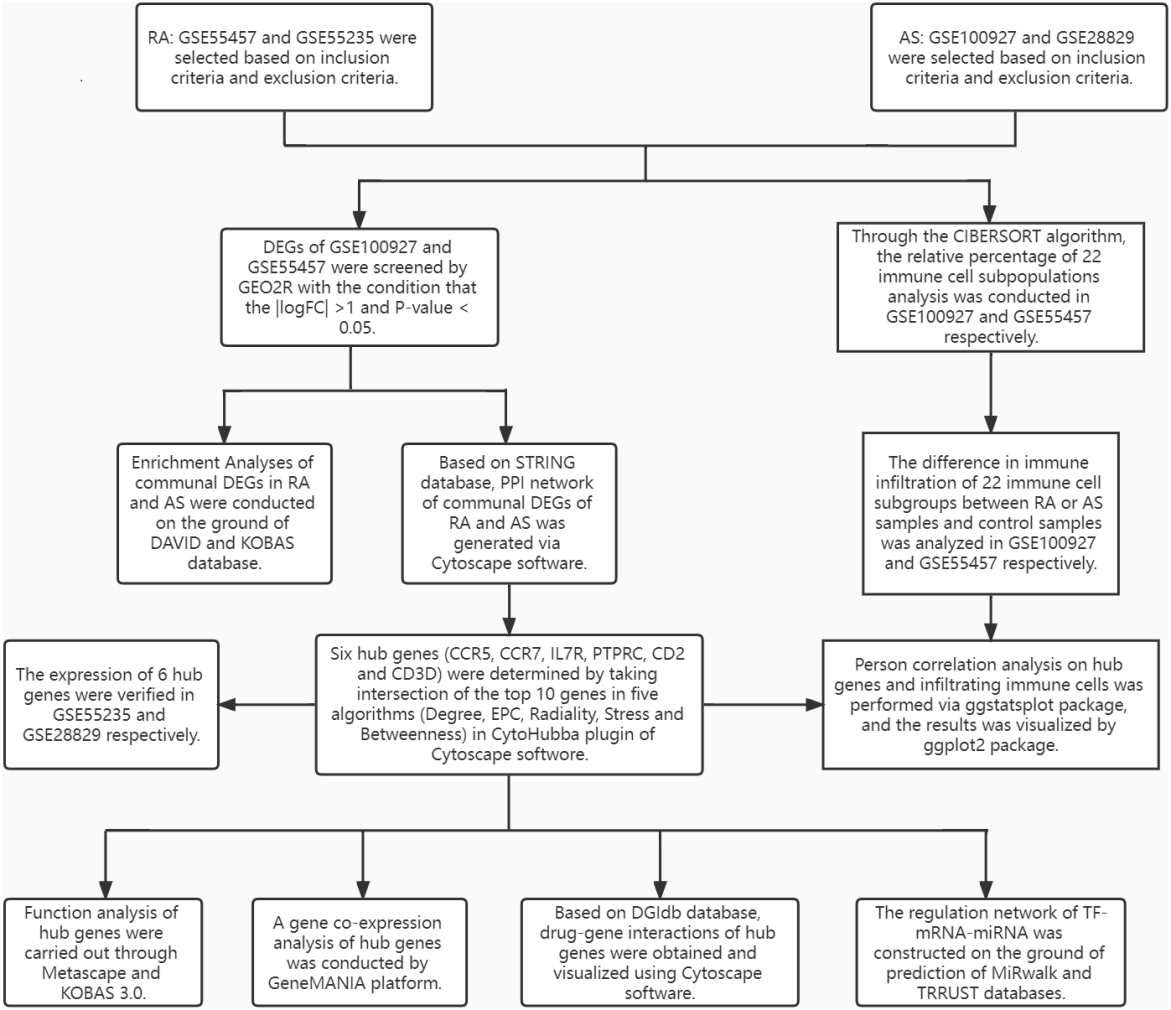
The research design flowchart.

**Figure 2:**
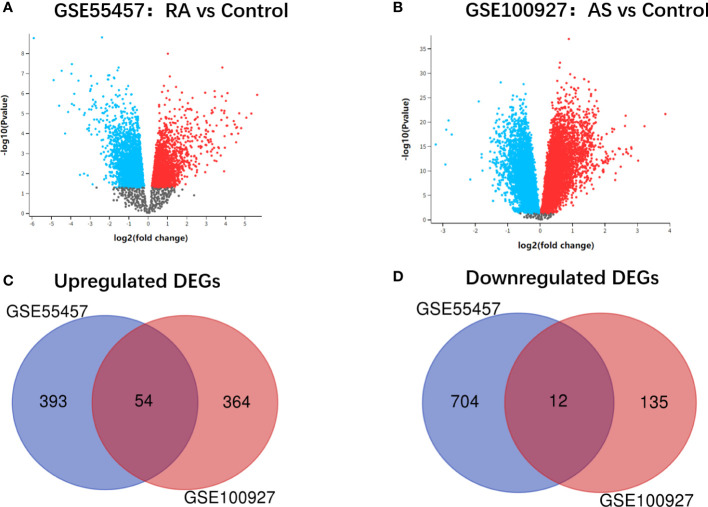
**(A)** Volcano plot of GSE55457. **(B)** Volcano plot of GSE100927. Upregulated genes are marked in red; downregulated genes are marked in bule. **(C)** Venn diagram of the 54 upregulated communal DEGs between RA and AS. **(D)** Venn diagram of the 12 downregulated communal DEGs between RA and AS.

The research design flow chart is shown in **Figure 1**. After standardizing the microarray results, DEGs of RA and AS were screened respectively. In the RA group, there were 1163 genes (447 upregulated and 716 downregulated genes) in GSE55457 identified as DEGs (**Figure 2A**). Meanwhile, 565 genes (418 upregulated and 147 downregulated genes) in GSE100927 were screened as DEGs in AS patients compared with controls (**Figure 2B**).After taking the intersection of the DEGs in GSE100927 and GSE55457, we obtained the communal DEGs (54 upregulated and 12 downregulated genes) between RA and AS (**Figures 2C, D**).

**Figure 3:**
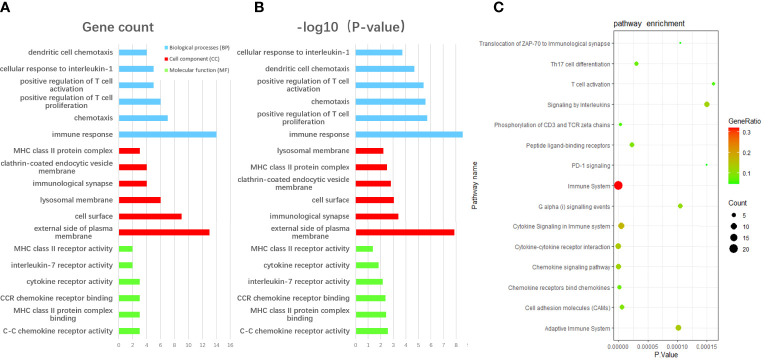
The enrichment analysis of communal DEGs between RA and AS. **(A)** The GO enrichment analyses of communal DEGs with gene count and **(B)** P-value based on the DAVID database. **(C)** The pathway enrichment analysis of communal DEGs on the ground of KOBAS 3.0. P-value < 0.05 was considered significant.

We performed GO enrichment analysis using the David database to unearth the biological characteristic of the communal DEGs, and the result was divided into three functional parts consisting of biological processes (BP), cell component (CC), and molecular function (MF) (**Figures 3A, B**). The enrichment results of the overlap DEGs are mainly ranked in terms of enrichment P-value in three functional parts respectively. In the BP category, DEGs were mainly enriched in immune response (GO:0006955), positive regulation of T cell proliferation (GO:0042102), chemotaxis (GO:0006935) and positive regulation of T cell activation (GO:0050870). Regarding the CC category, DEGs were significantly involved in external side of the plasma membrane (GO:0009897), immunological synapse (GO:0001772), cell surface (GO:0009986) and clathrin-coated endocytic vesicle membrane (GO: 0030669). As for the MF category, DEGs were significantly involved in C-C chemokine receptor activity (GO:0016493), MHC class II protein complex binding (GO:0023026), CCR chemokine receptor binding (GO:0048020) and interleukin-7 receptor activity (GO: 0004917). Moreover, according to KOBAS 3.0, the pathway enrichment analysis of overlapped DEGs showed that the genes were mainly enriched in the Immune System, Cytokine Signaling in the Immune system, Cytokine-cytokine receptor interaction(**Figure 3C**).

### PPI network construction and hub gene selection

**Figure 4:**
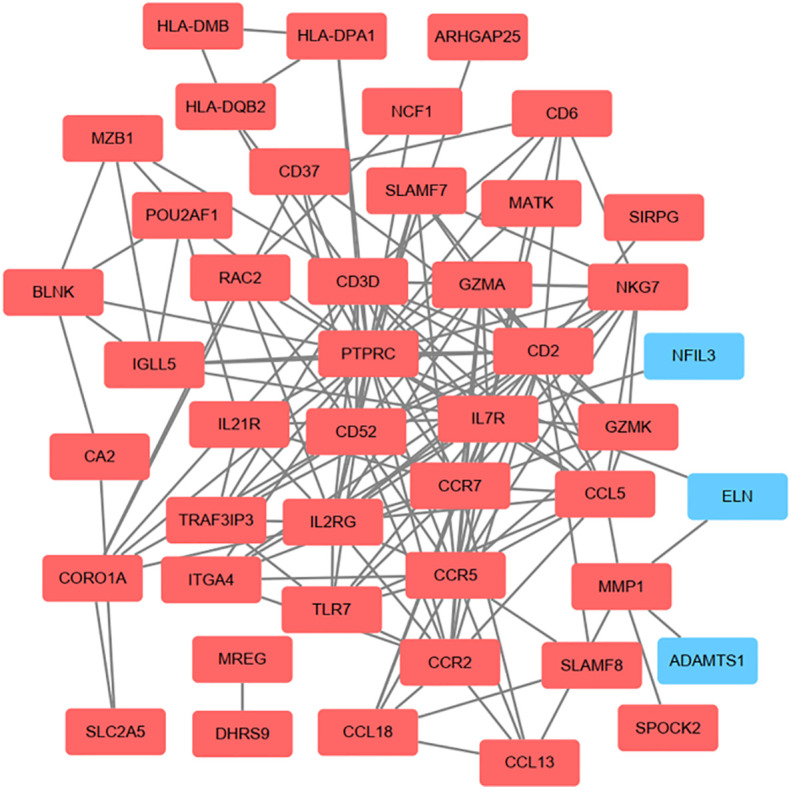
On the ground of STRING database, PPI networks of the communal DEGs were constructed by Cytoscape software. The red round rectangle represents upregulated genes and the blue round rectangle represents downregulated genes.

**Figure 5:**
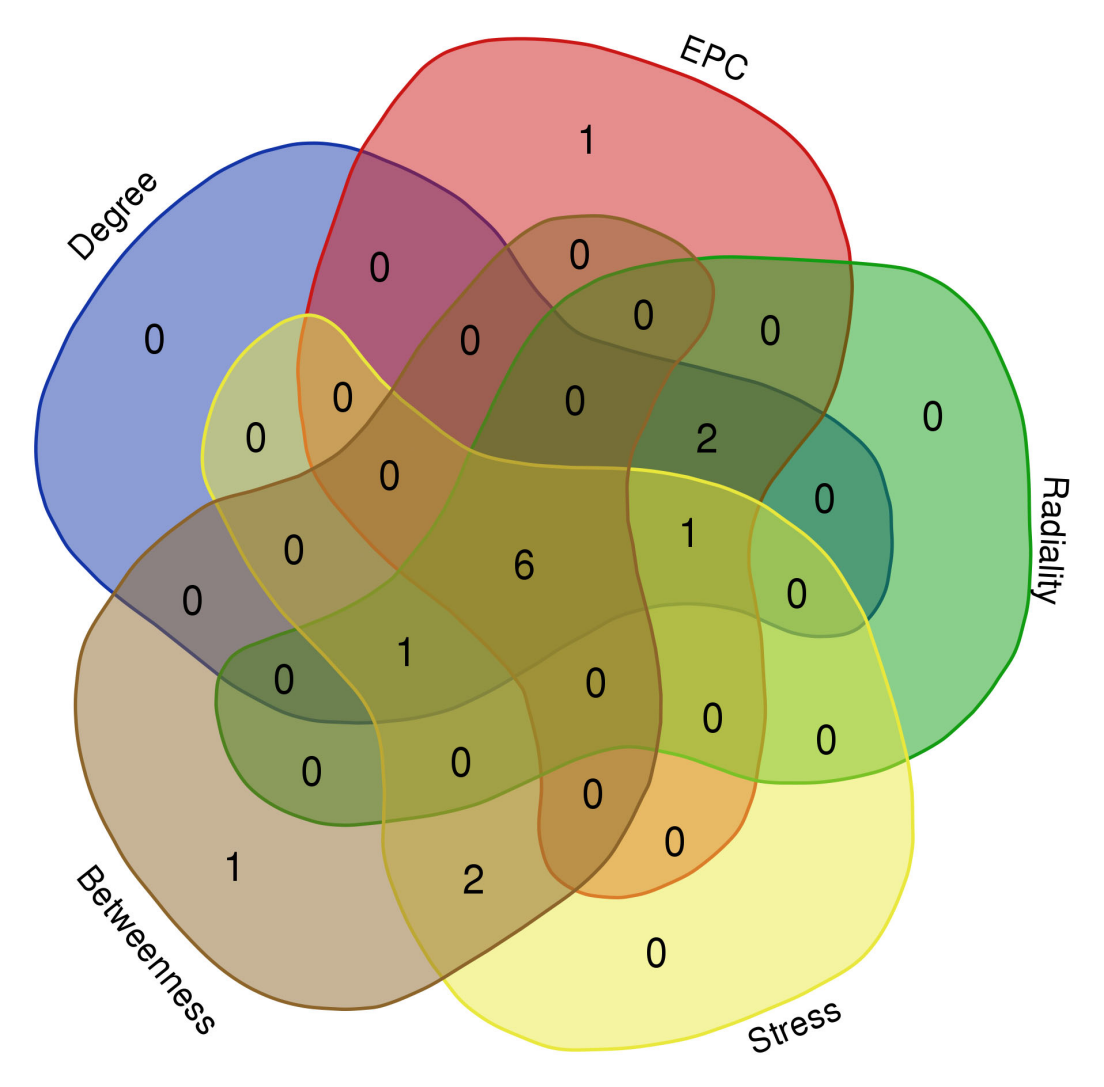
Six hub genes were determined by taking the intersection of the first 10 genes in the five algorithms of cytoHubba plugin (Degree, EPC, Radiality, Stress, and Betweenness).

On the ground of the STRING database, the PPI network of overlap DEGs was constructed using Cytoscape with the condition of combined scores of >0.4 points, consisting of 45 nodes and 162 edges (**Figure 4**). After that, five algorithms (Degree, EPC, Radiality, Stress, and Betweenness) in the CytoHubba plugin were selected to identify hub genes. The top 10 genes calculated by the five above-mentioned algorithms are listed in **Table 1**. Finally, by taking the intersection of the top 10 genes in five algorithms, six central genes (*CCR5, CCR7, IL7R, PTPRC, CD2*, and *CD3D*) were determined as hub genes (**Figure 5**). All six hub genes were upregulated genes.

**Table 1:** The top 10 genes in 5 algorithms.

Degree	EPC	Radiality	Stress	Betweenness
PTPRC	PTPRC	PTPRC	PTPRC	PTPRC
CD2	CD2	CD2	CD2	CD2
IL7R	IL2RG	IL7R	CCL5	MMP1
CCR5	IL7R	CD3D	IL7R	CCL5
CD3D	CCR5	IL2RG	CD3D	IL7R
IL2RG	CCR7	CCR7	MMP1	CD3D
CCR7	GZMA	CCR5	CCR5	CORO1A
CD52	CD3D	CCL5	CCR7	BLNK
CCL5	CD52	GZMA	IL2RG	CCR5
GZMA	NKG7	CD52	CORO1A	CCR7

### Hub gene analyses and validation

**Figure 6:**
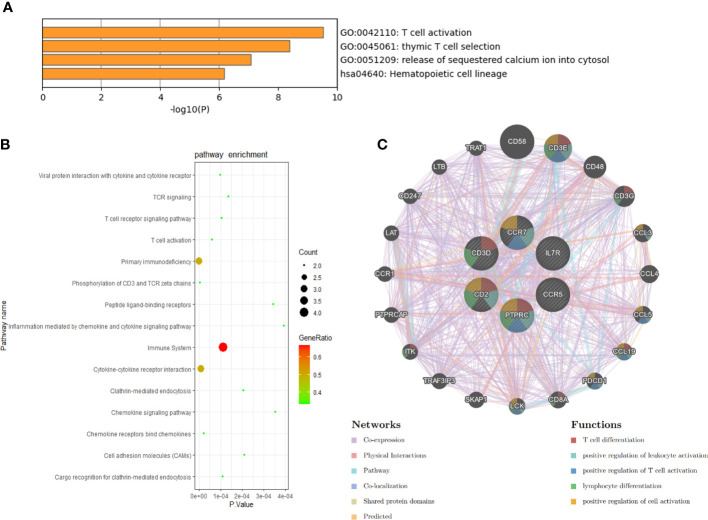
Biological process, pathway, and co-expression analysis of the hub genes. **(A)** The functional annotation analysis of hub genes *via* Metascape. **(B)** The further pathway enrichment analysis of hub genes by KOBAS 3.0. P-value < 0.05 was considered significant. **(C)** The network of hub genes and their co-expression genes were constructed and analyzed by GeneMANIA.

**Figure 7:**
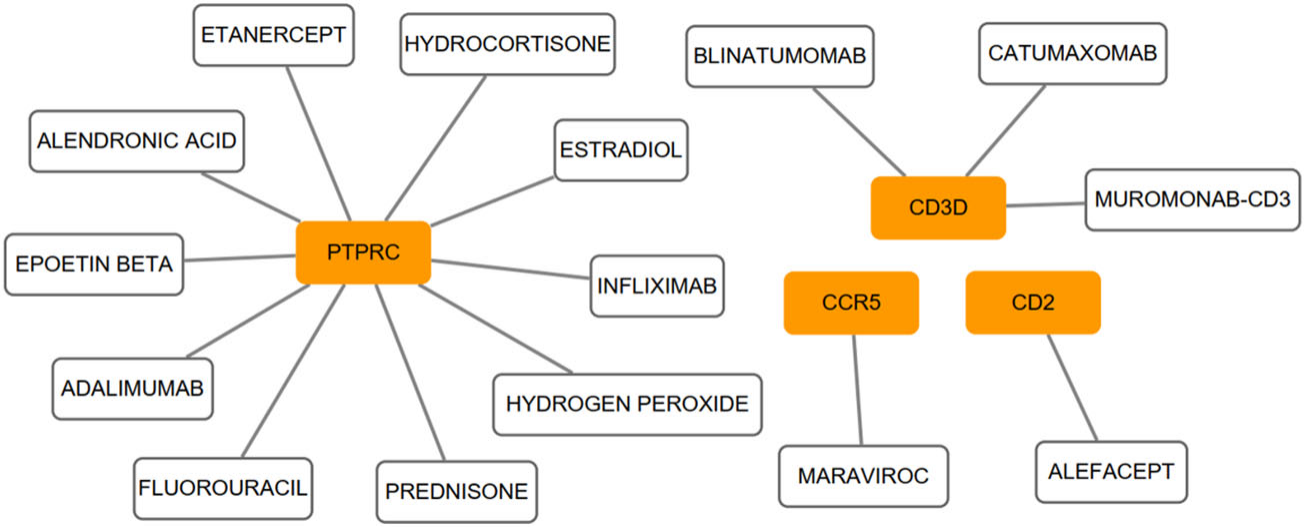
On the ground of the DGIdb database, the drug-gene pairs network was generated *via* Cytoscape, consisting of 4 hub genes (PTPRC, CD3D, CCR5 and CD2) and 15 drugs. Yellow circle indicates the hub gene and blank square indicates the drug.

We conducted the functional annotation analysis of hub genes *via* Metascape to better uncover the biological characteristics and mechanism of the six hub genes (**Figure 6A**). Hub genes were mainly involved in T cell activation (GO:0042110) and thymic T cell selection (GO:0045061). Besides, further pathway enrichment analysis was performed by KOBAS 3.0, and hub genes were significantly involved in the Immune System, Primary immunodeficiency, and Cytokine-Cytokine receptor interaction (**Figure 6B**). Similar to the analysis outcome of overlap DEGs, the gene annotation analysis revealed that hub genes were associated with the process of the immune system reacting. Subsequently, the network of the hub genes and their co-expression genes were generated on the ground of the GeneMANIA platform (**Figure 6C**). Six hub genes showed the complex PPI network with the Co-expression of 69.90%, Physical interactions of 10.20%, Pathway of 9.28%, Co-localization of 5.83%, Shared protein domains of 3.22% and Predicted of 1.57%. As expected, the biological function and roles of the hub genes re-emphasized the importance of the immune system in RA and AS. In addition, on the grounds of the DGIdb database, 15 drug-gene pairs were obtained with the condition that the drug should be FDA-approved, including 4 hub genes (PTPRC, CD3D, CCR5 and CD2) and 15 drugs. The categories of predicted drug are varied and all are FDA-approved drugs. Among them, there were a total of 10 potential drugs that could have effect on PTPRC, yet no drug was found to interact with multiple genes at the same time. These outcomes might reveal clues of potential therapeutic direction (**Figure 7**).

**Figure 8:**
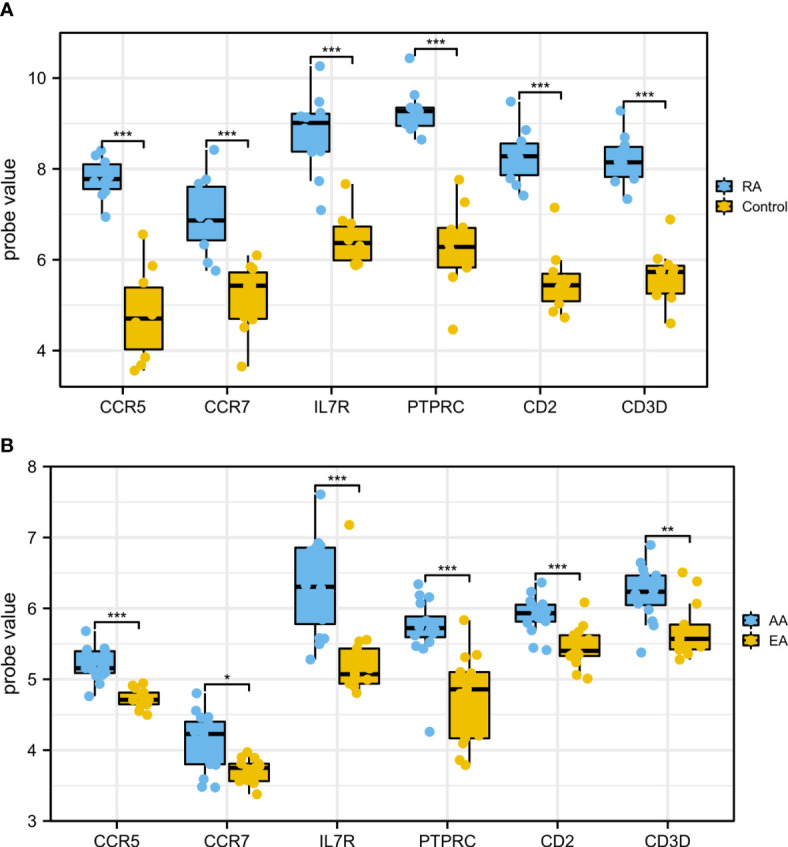
The expression of hub genes in **(A)** GSE55235 and **(B)** GSE28829. The comparison between the two datasets used the mean t-test. P < 0.05 was considered to be statistically significant. EA, early atherosclerotic plaques; AA, advanced atherosclerotic plaques. *P < 0.05; **P < 0.01; ***P < 0.001.

In order to ensure the reliability and accuracy of bioinformatics analysis results, GSE55235 and GSE28829 were adopted to verify the expression of hub genes in RA and AS samples by independence testing analysis respectively (**Figure 8**). Encouragingly, compared with controls, all 6 hub genes were significantly upregulated in AS and RA samples.

### Construction of the TF-mRNA–miRNA regulatory network

**Figure 9:**
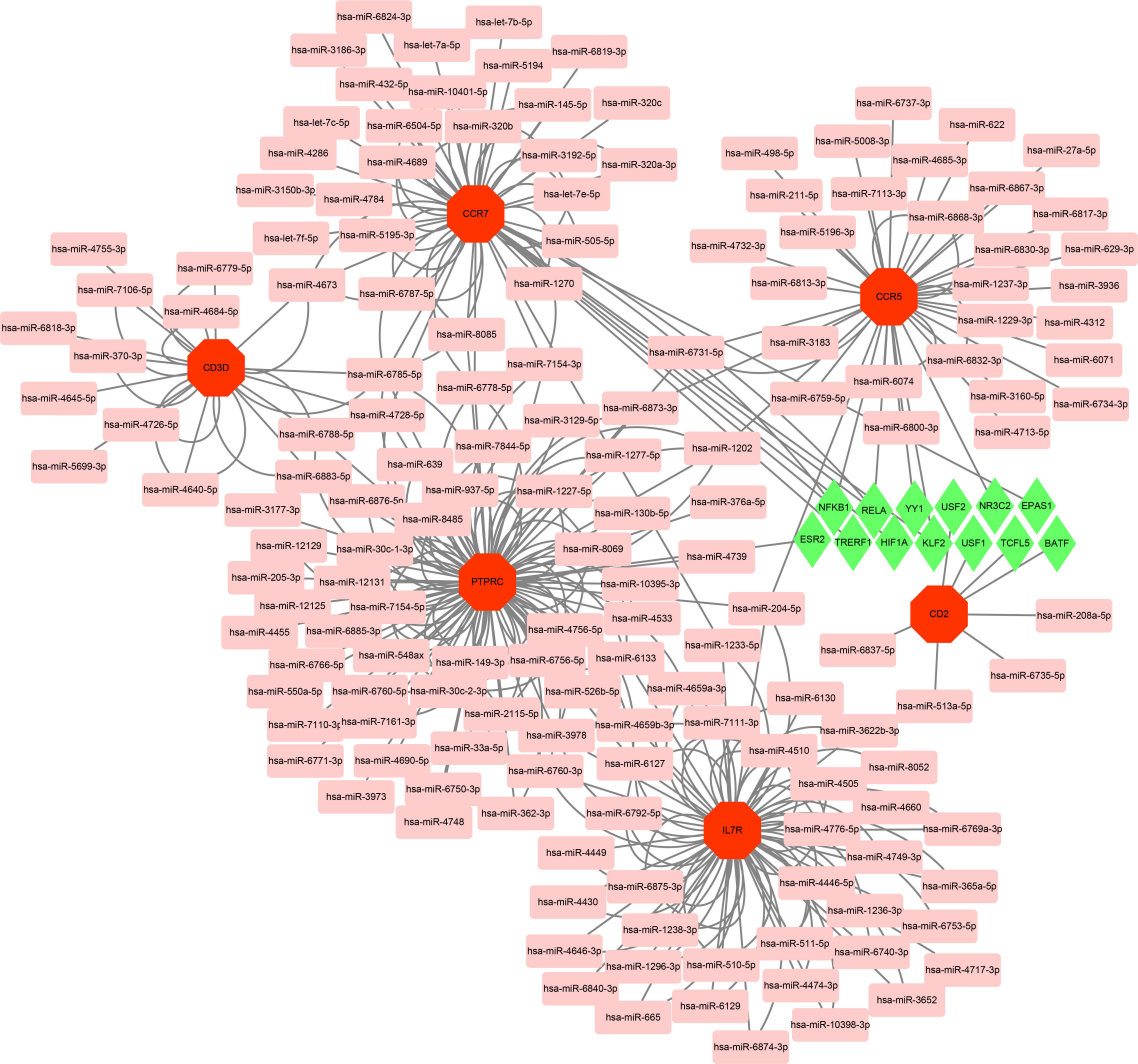
Based on MiRwalk and TRRUST database, TF-mRNA–miRNA regulation network of hub genes was constructed using Cytoscape. Hub genes were marked in red octagon; miRNAs were marked in pink round rectangle; TFs were marked in green diamond.

With the strict condition predicting that miRNA of hub genes could be verified by experiments or other databases, a total of 162 miRNAs were screened based on the MiRwalk database. Meanwhile, on the ground of the TRRUST database predictions of six hub genes, up to 13 TFs that could regulate hub genes were obtained. Then, based on the outcome of the prediction, the regulatory network of TF-mRNA-miRNA was constructed using Cytoscape software (**Figure 9**).

### Evaluation of immune cell infiltration

**Figure 10:**
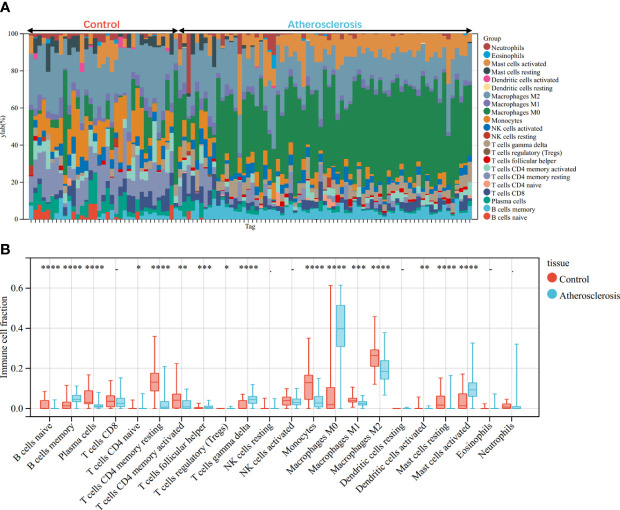
Immune cell infiltration mode in AS. **(A)** The relative percentage of 22 immune cell subpopulations out of 104 samples from the GSE100927. **(B)** The ratio of 22 immune cells between AS and control in GSE100927. *p < 0.05; **p < 0.01; ***p < 0.001; ****p < 0.0001.

**Figure 11:**
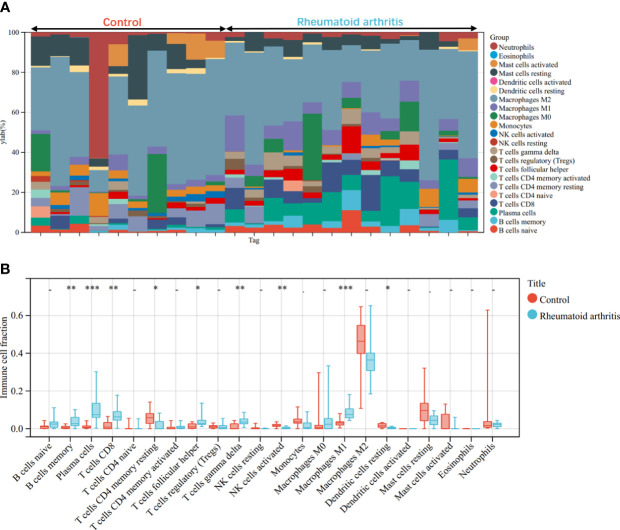
Immune cell infiltration mode in RA. **(A)**The relative percentage of 22 immune cell subpopulations out of 23 samples from the GSE55457. **(B)** The ratio of 22 immune cells between RA and control in GSE55457. *p < 0.05; **p < 0.01; ***p < 0.001.

Through the CIBERSORT algorithm, the relative percentage of 22 immune cell subpopulations analysis was conducted in GSE100927 and GSE55457 respectively (**Figures 10A**, **11A**). Subsequently, we analyzed 22 immune cell subgroups difference between disease samples and control samples. In terms of AS, there were more memory B cells, CD4+ naïve T cells, γδT cells, follicular helper T cells, M0 macrophages, and mast cells activated in AS tissue compared to control tissue, but fewer B naïve cells, plasma cells, CD4+ activated memory T cells, monocytes, M1 macrophages, M2 macrophages, activated dendritic cells, and resting mast cells (**Figure 10B**). As for RA, the violin chart showed that compared with the normal control sample, there were more memory B cells, plasma cells, CD8+ T cells, follicular helper T cells, γδT cells, and M1 macrophages in the RA samples, but fewer CD4+ resting memory T cells, activated NK cells, and resting dendritic cells (**Figure 11B**). Comprehensive analysis of immune infiltration outcome revealed that upregulated memory B cells, follicular helper T cells, and γδT cells could be the common immune process and mechanism between RA and AS.

### Correlation analysis of hub genes and infiltrating immune cells

**Figure 12:**
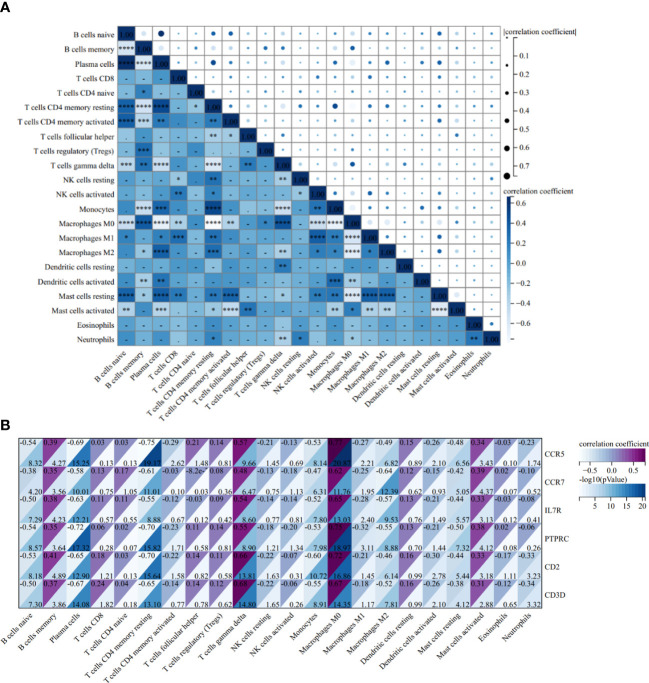
Correlation Analysis in AS. **(A)** Related heat maps of 22 immune cells in GSE100927. **(B)** Correlation heat map between hub genes and immune cells in GSE100927. *p < 0.05; **p < 0.01; ***p < 0.001, ****p < 0.0001.

**Figure 13:**
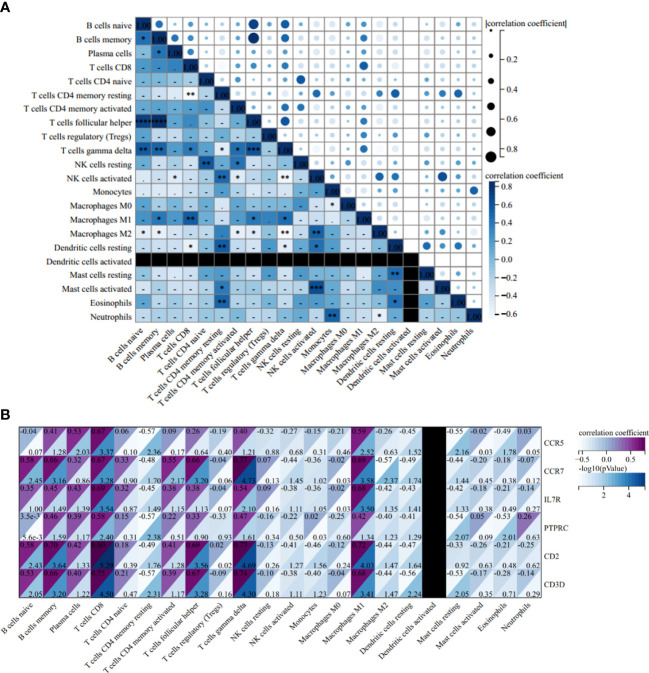
Correlation Analysis in RA. **(A)** Related heat maps of 22 immune cells in GSE55457. **(B)** Correlation heat map between hub genes and immune cells in GSE55457. *p < 0.05; **p < 0.01; ***p < 0.001.

The correlation heatmap of 22 types of immune cells showed that γδT cells had a significant positive correlation with follicular helper T cells and memory B cells, but the correlation between memory B cells and follicular helper T cells is statistically insignificant in AS (**Figure 12A**). As for RA, the correlations between γδT cells, follicular helper T cells and memory B cells were all significantly positive (**Figure 13A**).

In the AS group, the correlation analysis showed that all six hub genes were positively correlated with memory B cells and γδT cells, yet only CCR5 was positively correlated with follicular helper T cells. Additionally, and the correlation between other hub genes and follicular helper T cells was statistically insignificant (**Figure 12B**). As for RA, *CCR7, IL7R, PTPRC, CD2*, and *CD3D* were positively correlated with memory B cells and γδT cells, and *CCR7, CD2*, and *CD3D* were positively correlated with follicular helper T cells (**Figure 13B**).

## Discussion

In this study, we identified 54 upregulated and 12 downregulated communal DEGs in RA and AS. Enrichment analyses showed that these genes were significantly involved in the immune system and related immune signaling pathways. Subsequently, six hub genes (*CCR5, CCR7, IL7R, PTPRC, CD2*, and *CD3D*) were determined in the PPI network based on five algorithms (Degree, EPC, Radiality, Stress, and Betweenness) in the Cytohubba plugin. The function annotation analysis of hub genes and analysis of the co-expression network re-emphasized the importance of the immune system in RA and AS. Besides, we also predicted the drug-gene pairs and constructed the TF-mRNA-miRNA regulatory network to further elucidate the potential biological role of hub genes. Finally, the immune infiltration of RA and AS was analyzed and compared based on the CIBERSORT algorithm, and the correlation between hub genes and infiltrating immune cells was evaluated to reveal the relevant immune mechanisms.

Based on the results of the immune infiltrating landscape between AS and RA, we can dig out that the B memory cells, γδT cells, and follicular helper T cells were both higher in the disease group. Thus, we hypothesize that the pathogenesis between AS and RA shared some commonness, which was correlated with memory B cells and γδT cells. B cells play an essential role in RA pathogenesis. On the one hand, B cells can locally infiltrate the affected joints synovial membranes with autoantibodies, such as the rheumatoid factor (RF) and anti-cyclic citrullinated peptide (ACPA) (39). On the other hand, B cells serve as the antigen presentation cell, various inflammatory cytokines, and chemokines producers and CD4+T cells co-stimulator (40–42). However, B cells in the RA patient synovial tissue express memory B cell marker CD27 rather than naïve B-cell markers (43). Moreover, RA patients have an expansion of memory B cell subsets at the time of clinical episodes, which has been associated with worse long-term clinical outcomes (44). For the AS, both cellular and humoral B cell immunity play an important role in atherosclerotic plaque formation (45). Clinical studies have demonstrated a negative association between atherosclerotic outcomes and the unswitched memory B cells, which mainly express immunoglobulin (Ig)M antibodies. Interestingly, the switched memory cells mainly express IgG or IgA antibodies, and univariate analysis uncovered that serum level of IgG has been positively associated with atherosclerosis (46). Additionally, Hamze et al. has identified that B cells secreting IgG and IgA are present in the human atherosclerotic plaques located in the vascular wall (47). However, the characteristics of memory B cells in AS are intricacy, and memory B cells may participate in AS and RA development. Different from B memory cells, there was still lack direct evidence of the position and role of γδT cells in two diseases. γδT cells, a subtype of T cell, are different from αβ T cells because they cannot recognize specific antigens (48). Despite little evidence on the role of γδT cells in AS, γδT cells could modulate AS *via* IL-17 production owing to it being a rich source of IL-17. IL-17 was known related to favoring plaque stability (49), and increased IL-17 is one of the key molecules in RA pathogenesis (50). Notably, studies have shown that γδT17 cells are the main innate cellular source of IL-17 in collagen-induced arthritis models (51, 52). Meanwhile, the number of γδT17 cells is equal to regular T-helper 17 (Th17) cells in mice synovium, and the proportion of γδT17 cells in the joints has been more dramatically increased than Th17 cells (53). Thus, the mechanism of γδT cells between RA and AS remains unclear, and more basic and clinical research is needed to understand the mechanism. In addition, correlation analysis indicated that all 6 hub genes are positively correlated with at least one communal high-expressed immune cell in both diseases. Meanwhile, both the functional annotation analysis of hub genes and analysis of co-expression network point to the immune system and associated biological pathway. Based on the above analysis, we speculate that hub genes may act as mediator in associated immune pathways and affect immune cell action.

CCR5, a seven-transmembrane-spanning G protein-coupled receptor, is the chemokine receptor for CCL3, CCL4, CCL5, CCL8 and CCL3L1 (54, 55). Strikingly, although CCR5 is predominantly expressed on stimulated macrophages, it is also expressed on osteoclasts and vascular smooth muscle cells (56, 57). In RA pathogenesis, CCR5 induces osteoclast formation to influence osteoclast function. Clinically, CCR5 loss function decreased incidence and/or severity of human RA (58–62), and CCR5 intervention has been negatively related to human RA (63). In AS animal models, inhibiting CCR5 has been identified as a safeguard for lesion size, macrophage infiltration and plaque stability (54). In clinical practice, CCR5Δ32 polymorphism, causing a truncated nonfunction receptor, has been demonstrated as a protective influence on the risk of cardiovascular disease (64). Therefore, CCR5 may be the risk factor for both RA and AS and is involved in both RA and AS development. CC-chemokine receptor 7 (CCR7), also a G protein receptor, is the sole receptor for CCL19 and CCL21 (65). In RA, CCL21 and CCR7 are highly co-expressed and play a role throughout RA pathogenesis (66). CCR7 is overexpressed on RA dendritic cells, which has been closely related to levels of RF and C-reactive protein (67). What’s more, CCR7 expression is also elevated in RA synovial tissue macrophages, fibroblasts, and endothelial cells (66, 68). Intriguingly, activated CCR7 in these cells can directly or indirectly facilitate RA angiogenesis (69). Meanwhile, for AS, CD68+ macrophages elevate CCR7 expression-promoted low-density lipoprotein (LDL) binding and foam cell formation in both asymptomatic and symptomatic carotid plaques (70). CCR7 plays a critical part in cell migration; therefore, the deficiency of CCR7 reduces atherosclerotic plaque content and disturbs the T cells entry or exit in the inflamed vessel wall (71). Nevertheless, considering the pleiotropic nature of CCR7 in RA and AS, therapy targeting CCR7 would be a potential strategy to ameliorate both RA and AS.

IL-7R, a heterodimeric complex, is a receptor for IL-2, IL-4, IL-7, IL-9, IL-15, and IL-21. Pickens et al. have discovered that the proportion of M1 macrophages is increased in RA synovial fluid, with elevated IL-7R expression (72). IL-7 provokes RA-naïve myeloid cells to remodel into M1 macrophages, which are known as pro-inflammatory immune cells. Meanwhile, IL-7 induces osteoclast formation in M1 macrophages that express high IL-7R levels and are more responsive than naïve and M2 macrophages (73). Moreover, IL-7R ligation by IL-7 can also maintain T cell homeostasis and promote T cell proliferation, selection, activation, and cytokine production. Consistently, blocking IL-7 or IL-7R function can attenuate collagen-induced arthritis monocyte recruitment and osteoclast differentiation (74). Orchestrally, IL-7R antibody treatment in ApoE-/- mice has significantly reduced monocyte/macrophage cell infiltration and lipid content in the atherosclerotic plaque (75). Hence, the increased IL-7R expression may be required for AS and RA, and IL-R blocking would be valuable to hold back the development of AS and RA.

PTPRC, also known as CD45, encodes a protein that is a member of the protein tyrosine phosphatase (PTP) family. PTPs are known to be signaling molecules that regulate various cellular processes, and has been confirmed to be an essential regulator of T-cell and B-cell antigen receptor signaling (76, 77). Previous numerous studies have reported that PTPRC was densely associated with the anti-TNF therapy response in RA patients, meaning that PTPRC could effectively predict and guide personalized medicine in RA therapy (78–80). Consistent with our result, Xia et al. have demonstrated that PTPRC could act as a regulatory T cell (Treg)-related gene in the progression of atherosclerosis in previous bioinformatics analysis (81), yet there is still a lack of directly experiment evidence that PTPRC is correlated with atherosclerotic plaque evolution. The protein encoded by CD2 is a surface antigen found on all peripheral blood T cells. CD2 interacts with LFA3 (CD58) on antigen-presenting cells to optimize immune recognition (82, 83). Based on the GRAIL2 computational method, CD2/CD58 has been predicted to be new RA risk loci (84). Meanwhile, Fernandez Lahore G et al. found that the expression of CD2 was obviously upregulated in RA synovial tissue compared with osteoarthritis or healthy synovium and speculated that CD2 is densely involved in joint inflammation and CD2 polymorphisms because affecting its expression led to the development or perpetuation of joint autoimmunity (85). The interaction between CD2 and CD58 could promote T cells and macrophages to secrete chemokines and cytokines establishing the inflammatory environment, resulting in the formation of atherosclerotic plaque in psoriasis patients, yet the role of CD2/CD58 in the communal pathogenesis of RA and AS still lacks relevant evidence and needs to be further explored (86). CD3D encodes a part of the T-cell receptor/CD3 complex that takes part in T-cell development and signal transduction (87). Nowadays, for the association between CD3 and two diseases only bioinformatic analysis evidence exists, and more experiments are needed to uncover and verify the potential mechanism (88).

At present, drug treatment options for RA contains nonsteroidal anti-inflammatory drugs (NSAIDs), corticosteroids, disease-modifying antirheumatic drugs (DMARDs) and biologics (2, 89, 90). Cause the heterogeneous factors and complex pathological mechanisms in RA, there are still many patients have an unsatisfactory or poor clinical response under above therapy (90). In addition to new biologics and DMARDs with low side effects keeping in continuous developing states, some novel therapy directions such as GPCR-targeted drug and RNA therapeutics are positively explored and developed (89, 91). The potential therapy target of RA complicated with AS could lies in above explored directions. On the ground of our analyze outcome, the functional annotation analysis of DEGs and hub genes both indicated the role of chemokine receptors in two diseases. Chemokine receptors are a class of GPCRs that regulate immunity, and two above-mentioned hub genes (CCR5 and CCR7) are one type of this class (92). Currently, chemokine receptors are the most intensively studied GPCRs in RA, and the two main drug strategies of RA in chemokines and chemokine receptors as follow: corresponding ligands that can be selected to inhibit chemokine receptors and direct inhibition of chemokine receptors (1, 89). Because the chemokine is widely expressed in numerus human cells and the expression of chemokine might have diverse functional roles at different disease stages of RA, the development of GPCRs was still full of challenge (89). Nowadays, the main aspect of RNA therapeutics is RNA interference (RNAi). RNAi is an endogenous mechanism of mRNA silencing involving miRNA, and siRNA-based Interventions have revealed promising prospective in treatment of RA (93, 94). Based on the MiRwalk database and TRRUST database, the TF-mRNA–miRNA regulatory network of hub genes was predicted and constructed and might provide the potential exploring clues for RNA therapeutics.

The highlight of this article is to explore the communal immune pathway and identify the hub gene and immune infiltration profiles in RA and AS. However, our research also had some limitations as follows: First, although the sample size was slightly large and the hub gene was successfully verified in other gene datasets, this article is a retrospective study that still requires external verification to further verify findings. Second, the drug-genes interaction pairs and TF-mRNA–miRNA regulatory network could provide the primary clues for further exploring core genes, yet the exact and truly association and effect still needs more experiment to uncover. Third, the function of hub genes and immune cells needs to be validated and further explored in *in vitro* and *in vivo* models. The above-described experiments will be the focus of our future work.

## Conclusion

In summary, a total of 66 communal DEGs, six hub genes and three infiltrating immune cells were screened based on the comprehensive biological analysis. The evidence of overlapping pathogenesis between AS and RA points to relevant immune pathways, which might be mediated by specific hub genes and infiltrating immune cells. Furthermore, the analysis of these hub genes and immune cells may indicate new insights in potential therapeutic direction of RA complicated with AS.

## Data availability statement

The datasets presented in this study can be found in online repositories. The names of the repository/repositories and accession number(s) can be found in the article/**Supplementary Material**.

## Author contributions

All authors made substantial contributions to conception and design, acquisition of data, or analysis and interpretation of data; took part in drafting the article or revising it critically for important intellectual content; agreed to submit to the current journal; gave final approval of the version to be published; and agree to be accountable for all aspects of the work.

## Conflict of interest

Author WS was employed by China National Nuclear Corporation 416 Hospital.

The remaining authors declare that the research was conducted in the absence of any commercial or financial relationships that could be construed as a potential conflict of interest.

## Publisher’s note

All claims expressed in this article are solely those of the authors and do not necessarily represent those of their affiliated organizations, or those of the publisher, the editors and the reviewers. Any product that may be evaluated in this article, or claim that may be made by its manufacturer, is not guaranteed or endorsed by the publisher.
